# Neurotensin and its receptors mediate neuroendocrine transdifferentiation in prostate cancer

**DOI:** 10.1038/s41388-019-0750-5

**Published:** 2019-02-15

**Authors:** Shimiao Zhu, Hao Tian, Xiaodan Niu, Jiang Wang, Xing Li, Ning Jiang, Simeng Wen, Xuanrong Chen, Shancheng Ren, Chuanliang Xu, Chawnshang Chang, Amilcar Flores-Morales, Zhiqun Shang, Yinghao Sun, Yuanjie Niu

**Affiliations:** 1 Department of Urology, Tianjin Institute of Urology, The Second Hospital of Tianjin Medical University, 300211 Tianjin, China; 2 University of Minnesota, Minnesota, MN 55455 USA; 3 Department of Urology, Changhai Hospital, Second Military Medical University, 200433 Shanghai, China; 4 Department of Pathology, University of Rochester, Rochester, NY 14620 USA; 5 Department of Health Science, Faculty of Health and Medical Sciences, University of Copenhagen, 2200 Copenhagen, Denmark

## Abstract

Castration-resistant prostate cancer (CRPC) with neuroendocrine differentiation (NED) is a lethal disease for which effective therapies are urgently needed. The mechanism underlying development of CRPC with NED, however, remains largely uncharacterized. In this study, we explored and characterized the functional role of neurotensin (NTS) in cell line and animal models of CRPC with NED. NTS was acutely induced by androgen deprivation in animal models of prostate cancer (PCa) and activated downstream signaling leading to NED through activation of neurotensin receptor 1 (NTSR1) and neurotensin receptor 3 (NTSR3), but not neurotensin receptor 2 (NTSR2). Our findings also revealed the existence of a CK8^+^/CK14^+^ subpopulation in the LNCaP cell line that expresses high levels of both NTSR1 and NTSR3, and displays an enhanced susceptibility to develop neuroendocrine-like phenotypes upon treatment with NTS. More importantly, NTSR1 pathway inhibition prevented the development of NED and castration resistance in vivo. We propose a novel role of NTS in the development of CRPC with NED, and a possible strategy to prevent the onset of NED by targeting the NTS signaling pathway.

## Introduction

Prostate cancer (PCa) progression is largely dependent on androgen/androgen receptor (A/AR) signaling [1]. After a striking but temporary regression of tumors in the majority of patients undergoing androgen deprivation therapy (ADT), virtually all patients experience disease recurrence and progression to a disease state termed castration-resistant prostate cancer (CRPC) [2]. Drugs that further target A/AR signaling in CRPC, such as abiraterone and enzalutamide, have been approved for metastatic CRPC [3, 4]. Despite their successes, these novel agents for CRPC are similarly limited by primary and acquired resistance. In a significant number of cases, pathways independent of A/AR signaling emerge to sustain the development of CRPC [5].

Neuroendocrine (NE)-like PCa is identified by increased staining of neuroendocrine differentiation (NED) markers, e.g. neuron-specific enolase (NSE), chromogranin A (CgA), and synaptophysin (Syn), and is believed to originate from trans-differentiated tumor cells that give rise to CRPC following ADT [6, 7]. Markers of NED may be detected in up to 70% of PCa that have undergone ADT for more than 13 months, but rarely detected in ADT naïve tumors [8, 9], supporting the notion that ADT induces NED in PCa. NE-like PCa is highly aggressive, with only 35% 2-year survival rate [10, 11]. Since NE-like PCa is independent of A/AR signaling [5], alternative critical signaling pathways driving NED in CRPC must be identified and characterized in order to formulate effective strategies for novel therapeutic development.

A transcriptome analysis of archived tumor specimens revealed that NE-like PCa cells are more similar to non-NE PCa cells than normal NE cells [12], suggesting NE-like PCa cells originated from cancerous epitheliums through the trans-differentiation process [13–15]. Generally, there are three subgroups of epithelial cells in PCa: CK8^+^ luminal cells, CK14^+^ basal cells and CK8^+^/CK14^+^ intermediate cells. The specific cell type that contribute to the emergence of NE-like tumor cells after ADT remains unknown, although the composition and dynamics of each cell type may vary in different disease stages after ADT.

Neurotensin (NTS) has been implicated in NED. Other than the brain cells, NTS is widely expressed in various tissues. The function of NTS is mediated through the interaction with its receptors neurotensin receptor 1 (NTSR1), neurotensin receptor 2 (NTSR2), and neurotensin receptor 3 (NTSR3) [16]. NTS has been identified an as a NED marker [17], and it has demonstrated increased expression after castration [18]. Previous studies revealed an association of NTS with progression and invasiveness of PCa [19, 20], also offer preclinical proof for targeting the NTSR1 receptor as a potential pharmacological target in cancer therapy [19, 21]. However, an integrated understanding of acquired NED in the context of castration is lacking. Such insights might be used for designing more effective therapies to overcome resistance and improve survival from palliative to curative measures in PCa. In this study, we delineated how NTS induces NED development after ADT in cell line and animal models of CRPC with NED. Blocking NTS signaling with NTSR1 inhibitor in combination with ADT markedly delayed NED development and reduced tumor burden in preclinical models.

## Results

### NTS expression is elevated in CRPC xenografts and correlated with NE transdifferentiation

**Fig. 1:**
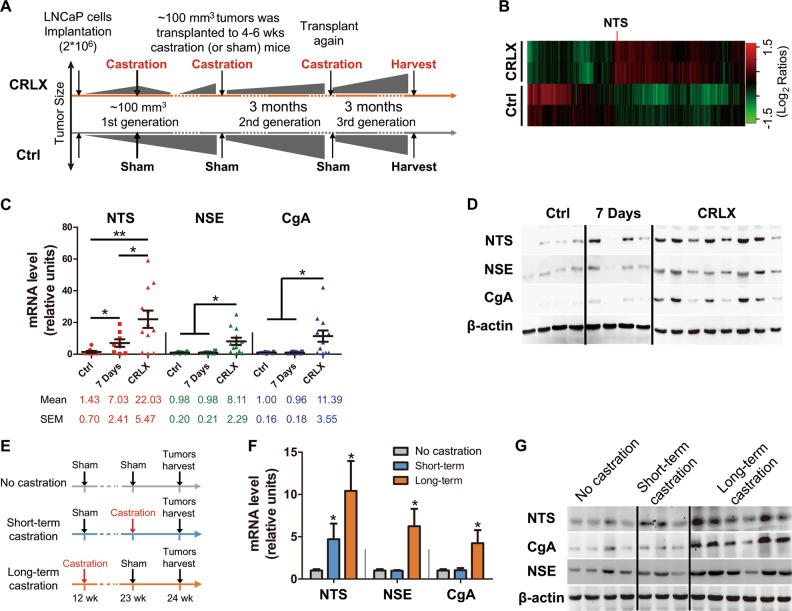
NTS expression is elevated in castration-resistant prostate cancer xenografts and correlated with neuroendocrine transdifferentiation. **a** Schematic illustrating the design of LNCaP mouse xenograft experiments. LNCaP cells were subcutaneously injected into BALB/c nude mice. Until tumors grew reached a size of ~100 mm^3^, xenografted mice were randomized to two groups: (1) castration-resistant LNCaP xenograft (CRLX), and control (Ctrl). Mice in CRLX group were castrated, while mice in control group received sham treatment until the first generation tumors were harvested after 2 months. Achieved tumors from above model were cut and minced to ~1 mm^3^, which were integrated to the size of about 100 mm^3^ by using matrigel and subcutaneously transplanted into the next generation of syngeneic mice. Repeating the circle process, the status of castration-resistant was determined until tumor in CRLX group grew faster than their reference. **b** Most differentially expressed genes in a cohort of CRLX (*n* = 8) compared to control (*n* = 4) determined by expression microarray analysis (Human Genome U133 Plus 2.0). The candidate genes were defined as fold change (CRLX vs. Ctrl) > 2 and false discovery rates (FDR) < 0.05. see also Supplementary Table 2. **c** qRT-PCR analysis of NTS, NSE, and CgA mRNA expression in a validation cohort of LNCaP tumors from mice treated with sham (control, *n* = 6), 7 days of castration (*n* = 6), or with acquired resistance to castration (*n* = 12). GAPDH used for normalization. **d** Western blot analysis of NTS, NSE, and CgA protein expression in a validation cohort of LNCaP tumors from mice treated with sham (control, *n* = 4), 7 days of castration (*n* = 4), or with acquired resistance to castration (*n* = 8). CRLX samples were loaded for analysis from high to low NTS levels based on mRNA analysis. **e** Schematic illustrating the design of TRAMP mice experiments. Mice were divided into three groups followed by receiving sham, short-term, or long-term surgical castration, then tumors were harvested at the same time. **f** qRT-PCR analysis of NTS, NSE, and CgA mRNA expression in primary tumors from uncastrated (*n* = 4), short-term (*n* = 3), and long-term (*n* = 6) castrated TRAMP mice. GAPDH used for normalization. **g** Western blot analysis of NTS, NSE, and CgA protein expression in a subset of tissues from uncastrated (*n* = 4), short-term (*n* = 3), and long-term (*n* = 6) castrated TRAMP mice. ^*^p < 0.05, ^**^p < 0.01

We have previously shown that castration-resistant LNCaP xenografts (CRLX) could be established and serially transplanted under castrate conditions according to the schema shown in Fig. 1a [22]. Aggregated microarray data from CRLX and control tumors were compared to identify differentially expressed genes associated with resistance (Fig. 1b and Supplementary Table 2). The most upregulated genes in the CRLX were the Hyaluronan and proteoglycan link protein 1 (HAPLN1), Keratin 13 (KRT13), Fibrillin 1 (FBN1), Aldehyde dehydrogenase 1 family member A1 (ALDH1A1), and neurotension (NTS) (Supplementary Table 2). As a NED marker, NTS were noticed. Elevated mRNA expression of NTS was validated by quantitative RT-PCR (Fig. 1c) comparing established CRLX specimens with castration-naïve specimens. Interestingly, elevated NTS expression was also observed at 7 days after castration (Fig. 1c). Although concomitant elevation of NSE and CGA, traditional NED markers, were demonstrated in CRLX specimens, these markers were not elevated at 7 days after castration (Fig. 1c), suggesting elevation of NTS occurs earlier than other NED markers after castration. Western blot analysis in a subset of samples (with sufficient protein lysates) confirmed this observation (Fig. 1d).

To determine whether NTS elevation following castration can be observed in a different in vivo PCa model, we conducted experiments in the TRAMP mice according to the schema shown in Fig. 1e. The earlier elevation of NTS after short-term castration (one week after castration) and positive correlation between NTS and NED after long-term castration were further confirmed in the TRAMP mouse model (Fig. 1f and g). Together, these findings suggest that NTS may be acutely induced by castration in vivo, and elevated NTS may be required for the establishment of the NED phenotype.

### NTS is sufficient to induce NED in vitro

**Fig. 2:**
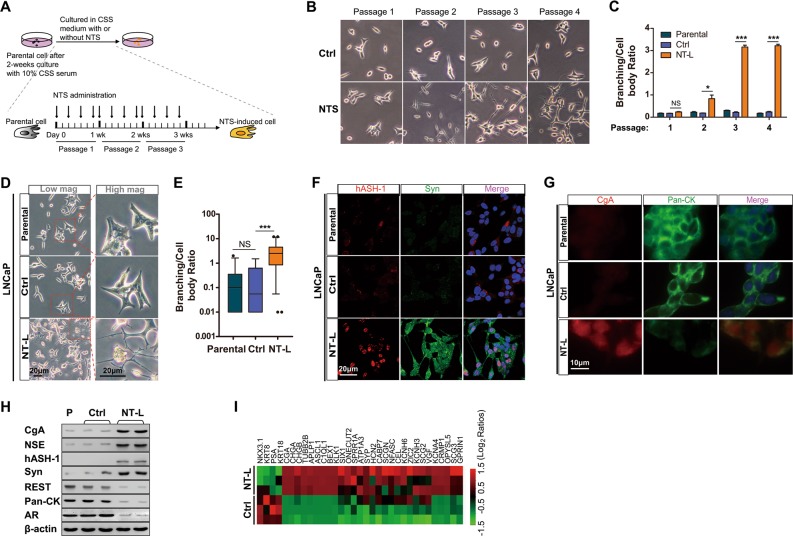
NTS induces neuroendocrine differentiation in prostate cancer cell lines. **a** Schematic illustration of the procedure inducing NE-like cells from prostate cancer cell lines. Before NTS treatment, cells were cultured in 1640 medium with CSS serum (charcoal stripped serum) for 2 weeks to mimic the castration conditions. Then, cells were induced by NTS or DMSO for 3 weeks (LNCaP cells) or 5 weeks (C4-2 cells). **b** Representative cell images in bright field of each group from different passages. **c** Quantification of branching/cell body ratio in each group from different passages. Branching/cell body ratio was determined for three microscopy fields, *n* = 171–242 total cell count. *P* values were evaluated by Mann–Whitney *U* test. **d** Bright field microscopy showed the cell morphology of parental, DMSO-treated (Ctrl), and NTS (4 ng/ml)-treated LNCaP cells (NT-L) on passage 3. Mag: magnification. **e** Branching/cell body ratio of individual cells from each group shown in (**b**) was calculated. *P* values were evaluated by Mann–Whitney *U* test. **f** Immunofluorescence double staining of helix-loop-helix transcription factors (hASH1) and synaptophysin (Syn) shows the NE-like phenotype in passage 3NT-L. **g** Immunofluorescence double staining of chromogranin A (CgA) and pan-cytokeratin (pan-CK) showed increased neuroendocrine marker and decreased epithelial cytokeratin marker in passage 3NT-L. **h** The differential expression of CgA, NSE, hASH1, Syn, and pan-CK in parental, DMSO-treated control, and passage 3NT-L groups was shown in Western blot assay. **i** Heat map representing expression changes in NED-related genes [23] in control and NT-L cells. Heat map was generated by conversion of qRT-PCR data that was normalized to β-actin and β2M. ^*^p < 0.05, ^**^p < 0.01, ^***^p < 0.001. See also Supplementary Fig. 1

A series of in vitro experiments were carried out to test whether exogenous NTS is sufficient to induce NED. First, we determined that in vitro treatment with 4 ng/ml NTS could mimic endogenous signals in CRLX, on the basis of Erk1/2 response [19] (Supplemental Fig. 1a, b) and global gene signature analysis (Supplemental Fig. 1c, d). With the optimal NTS concentration established for in vitro experiments, we modeled NTS effects by subjecting LNCaP cells preconditioned by charcoal-stripped serum (CSS) to NTS treatment (Fig. 2a). Branching and extension of neuron-like processes, morphological features of NED, were evident at passage 3 in the NTS-treated LNCaP cells (NT-L) (Fig. 2b–e). In addition, NT-L cells expressed traditional NED markers as shown by both IF (Fig. 2f, g) and Western blot analysis (Fig. 2h, and Supplemental Fig. 1e). Moreover, NT-L cells displayed NED expression profile defined in a previous study [23] (Fig. 2g). Morphological features of NED were also evident at passage 5 in the NTS-treated C4-2 cells (NT-C) (Supplemental Fig. 2f), in which the molecular signature of NED was replicated (data not shown).

### NTS induces NED through NTSR1 and NTSR3

**Fig. 3:**
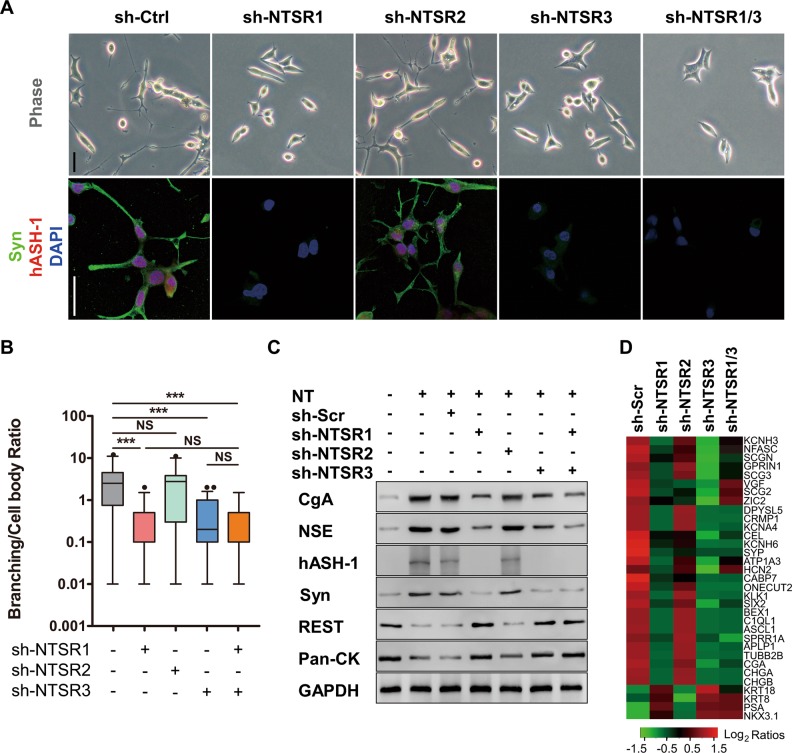
NTSR1 and NTSR3 are required for NTS-stimulated NED in LNCaP cells. **a** LNCaP cells, which were transfected with indicated shRNA to NTSR1, 2, 3, 1+3 and shRNA control, were treated with NTS (4 ng/ml). The NE phenotypes were shown in bright field microscopy (upper panel) and in immunofluorescence assay of Syn/hASH1 double staining (lower panel). **b** Branching/cell body ratio of cells shown in (**a**) was quantified and plotted as box and whisker plots (branching/cell body ratio was determined for three microscopy fields, *n* = 97–125 total cell count). *P* values were evaluated by Mann–Whitney *U* test. ^***^p < 0.001. **c** Immunoblotting analysis of CgA, NSE and Syn, HASH1 and pan-CK expression in control, NTS-treated, and following NTSR1, 2 and 3 knockdown groups. **d** Heat map representing expression changes in NED-related genes in NTS (4 ng/ml)-treated LNCaP cells with NTSR1, 2 and 3 knockdown by indicated shRNAs. Heat map generated by conversion of qRT-PCR data that was normalized to β-actin and β2M. Data are representative of two independent experiments. See also Supplementary Fig. 2

NTS action is mediated by three receptors, NTSR1, NTSR2, and NTSR3. NTSR1 and NTSR2 are G protein-coupled receptors, with high and low affinity NTS binding, respectively, while NTSR3 is a non-specific single trans-membrane-sorting receptor [24, 25]. We used shRNA to stably knockdown NTSR1, NTSR2, and NTSR3 in LNCaP cells (Supplementary Fig. 2). Down-regulation of NTSR1 and NTSR3, but not NTSR2, suppressed the NED phenotype induced by NTS in LNCaP cells (Fig. 3a–d). These findings demonstrated that NTSR1 and NTSR3, but not NTSR2, were required for NED in response to NTS stimulation.

### NTS targets CK8^+^/CK14^+^ cells

**Fig. 4:**
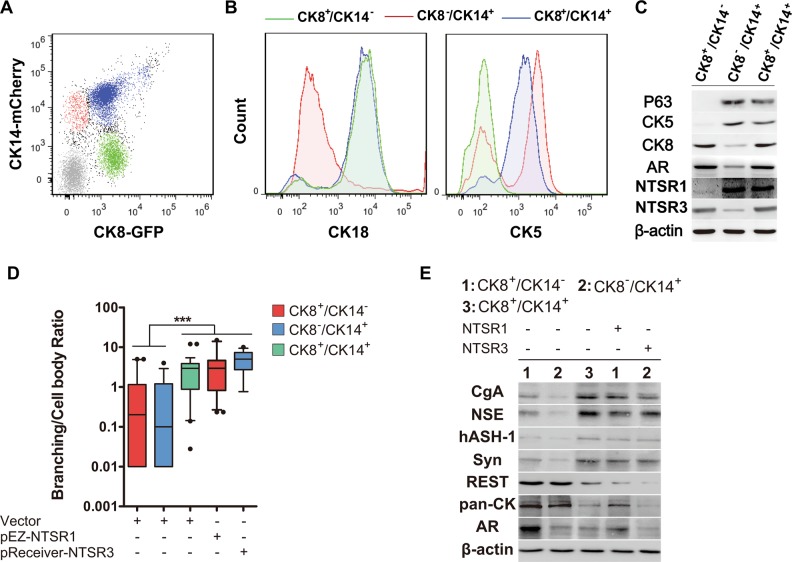
Characterization of the CK8^+^/CK14^+^ cell population as the origin of NE-like cells. **a** FACS plots showed the distribution of LNCaP cells, based on expression of CK14 and CK8 and gates drawn to distinguish three populations. Cells were transfected with CK8-eGFP and CK14-mCherry, and three populations of CK8^+^/CK14^−^, CK8^−^/CK14^+^, and CK8^+^/CK14^+^ were separated. **b** Expression of CK18 and CK5 in the three cell populations distinguished above. **c** Characterization of the three cell populations distinguished in (**b**) by immunoblotting analysis of basal markers, CK5 and p63, and luminal markers, CK8 and AR. NTSR1 and NTSR3 were also determined. **d** Branching/cell body ratio was calculated in indicated cells received treatment of NTS (4 ng/ml) for 3 weeks. Branching/cell body ratio was determined in three microscopy fields, *n* = 112–146 total cell count. *P* values were evaluated by Mann–Whitney *U* test. **e** Immunoblotting analysis of CgA, NSE, Syn, hASH1, AR, and pan-CK in indicated cells received treatment of NTS (4 ng/ml) for 3 weeks. ^***^p < 0.001. See also Supplementary Fig. 3

We have observed that although NED could be induced by NTS, not all cells demonstrate the NE-like phenotype [26] (Fig. 2). This observation likely suggested differential sensitivity to NTS in the cells exposed to NTS treatment. To identify the target cells for NTS, we designed the luminal keratin CK8 and basal keratin CK14 promoter reporter constructs (GLuc-ON™), in which the CK8 and CK14 promoters separately drive eGFP (green) and mCherry (red) expression (Supplemental Fig. 3a). Using this promoter reporter system, CK8^−^/CK14^+^, CK8^+^/CK14^−^, and CK8^+^/CK14^+^ cell subpopulations were sorted by FACS (Fig. 4a). Phenotypes of these sorted cell fractions were further analyzed by Western blotting and FACS (Fig. 4b, c), confirming that CK8^+^/CK14^−^ cells presented biomarkers of luminal cells, CK8^−^/CK14^+^ cells presented basal cell markers, and CK8^+^/CK14^+^ cells expressed both basal and luminal markers like intermediate cells. Interestingly, the intermediate-like cells expressed high levels of both NTSR1 and NTSR3, while lower level of NTSR1 and NTSR3 was, respectively, detected in CK8^+^/CK14^−^ and CK8^−^/CK14^+^ cells (Fig. 4c). Because both NTSR1 and NTSR3 are necessary for NED (Fig. 3), we hypothesized that intermediate-like cells are the target cells for NTS action. Indeed, after subjecting CK8^+^/CK14^−^, CK8^−^/CK14^+^, and CK8^+^/CK14^+^ cells to 4 ng/ml NTS, NED morphology (Fig. 4d) and NED markers (Fig. 4e) could only be detected in CK8^+^/CK14^+^ cells at passage 3 after NTS treatment (Fig. 4d, e). To determine whether expression of both NTSR1 and NTSR3 enables NTS response CK8^+^/CK14^−^ (NTSR1 negative) and CK8^−^/CK14^+^ cells (low NTSR3), the CK8^+^/CK14^−^ and CK8^−^/CK14^+^ cells were transfected with NTSR1 and NTSR3, respectively (Supplemental Fig. 3b, c). The transfected CK8^+^/CK14^−^ and CK8^−^/CK14^+^ cells with both receptors developed NED morphologies with increased NED markers at levels similar to those detected in CK8^+^/CK14^+^ cells (Fig. 4d, e).

### Suppression of NED and castration resistance by NTSR1 antagonist

**Fig. 5:**
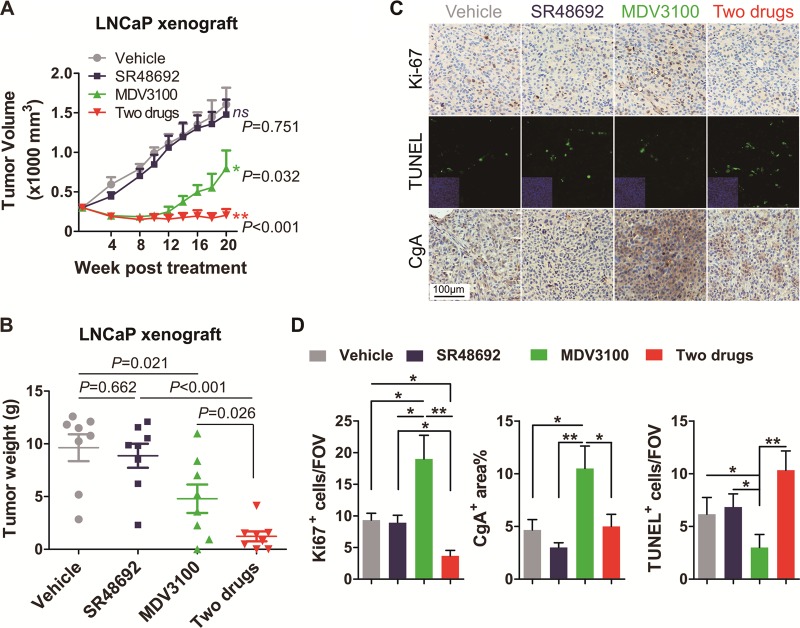
Suppression of NED and castration resistance by NTSR1 antagonist. **a** LNCaP cells were injected subcutaneously in mice and grown until tumors reached a size of ~300 mm^3^ Xenografted mice were randomized and then, received PEG vehicle, 25 mg kg^−1^ SR48692, 10 mg kg^−1^ MDV3100, or SR48692+MDV3100 for 5 days a week. Caliper measurements were taken biweekly. *n* = 8 mice per group. *P* values were determined by comparing with control group. **b** Individual tumor weight in each group is shown. **c** LNCaP cells were injected subcutaneously in mice and grown until tumors reached a size of ~300 mm^3^ Xenografted mice were randomized and then, received PEG vehicle, 25 mg kg^−1^ SR48692, 10 mg kg^−1^ MDV3100, or SR48692+MDV3100 for 5 days a week. Representative images of Ki-67, TUNEL, and CgA in LNCaP xenograft tumors after different therapy, specimens were got at 20 weeks post treatment. **d** Quantitation of Ki-67, TUNEL, and CgA expressions in LNCaP xenograft tumors from each group, specimens were got at 20 weeks post treatment. Immunostained area/FOV (per field of view) was quantified using ImageJ. *p* values were evaluated by Mann–Whitney *U* test. ^*^p < 0.05, ^**^p < 0.01. See also Supplementary Fig. 4

To determine the feasibility of suppressing NED in vivo, we evaluated a NTSR1 antagonist, SR48692, in LNCaP xenografts and TRAMP tumors. The mice were randomized and treated with vehicle, SR48692, enzalutamide, or the combination of enzalutamide and SR48692. Xenograft-bearing mice started to receive treatments until tumors reached size of ~300 mm [3], and TRAMP mice were treated at 12-week old [27]. In LNCaP Xenografts, Enzalutamide as a single agent significantly suppressed tumor growth initially, but tumor growth accelerated with increased NED after a period of therapy (Fig. 5a–d). Although SR48692 alone did not suppress NED and tumor growth, a remarkable reduction in NED and tumor growth was observed when it was combined with Enzalutamide (Fig. 5a–d). These findings were largely replicated in the TRAMP mice (Supplemental Fig. 4) Together, these results highlight the potential of targeting NTS signaling to delay or prevent the progression of CRPC through NED.

## Discussion

CRPC can arise through a variety of mechanisms, including AR gene mutations and amplification [28], bypass of AR pathway activation [29], activation of PCa stem cell niches [30, 31], and NE-like trans-differentiation [32]. NeCRPC is an aggressive subtype of CRPC raised after ADT [8]. However, the mechanism of trans-differentiation from PCa epithelial cells to NE-like cells is still not fully comprehended. In this study, we found that NTS could induce tumor cells trans-differentiation into NE-like cells through NTS-NTSR1/3 signaling. NTSR1 pathway inhibition prevented the development of NED and castration resistance in vivo. These findings are consistently replicated in multiple cell line and animal models of CRPC, suggesting the critical importance of NTS in mediating NED. With respect to the source of NTS in vivo, it is highly plausible that non-tumor cells in the tumor microenvironment may play a critical role [33]. Data in this paper supported that NTS function as a mediator directing tumor cell NED. However, the contribution of the tumor microenvironment as a plausible source of NTS is still under extensive investigation.

The NE-like cells in CRPC may be different from normal prostate epithelial NE cells [7]. The remnant cytokeratin in these NE-like cells [34] suggest they may originate from cancerous epithelial cells through a trans-differentiation process. The epithelial lineage from which NE-like cells arise remains controversial. Some suggested that the origin of malignant NE-like cells should be luminal cells due to their double-positive staining of luminal-markers and NE-markers [34]. Others postulated that NE-like cells derived from stem cells, due to co-expression of NE-markers with CD44 in human PCa tissues [35]. Our study provided direct evidence supporting that CK14^+^/CK8^+^ cells, which express both luminal markers and basal markers (including CD44) are the most likely candidates in NTS-induced NED. Figure 4a showing that there is a large population of double-negative cells, which seems caused by the imperfect transfection efficiency. It may cause misidentification some CK14^+^/CK8^+^ as CK14^+^/CK8^−^ or CK14^−^/CK8^+^ cells. So, it is unclear whether the entire CK14^+^/CK8^+^ subpopulation shares the potential to trans-differentiate into NE-like cells. Maybe the potential is carried by a smaller subgroup within CK14^+^/CK8^+^ cells. However, the imperfection does not weaken our conclusions that CK14^+^/CK8^+^ cells are the most likely candidates of NE-like cells.

Currently, there is no standard treatment for patients with NeCRPC, possibly reflecting a poor understanding of the mechanism driving the development of these tumors. Based on our data showing the biologic roles of NTS in NeCRPC, we propose a therapeutic strategy targeting NTS signaling to prevent NED. Suppression of NTS-NTSR1 signaling in LNCaP xenografts by NTSR1 inhibitor resulted in dramatically decreased NED and synergized with AR-targeting agent enzalutamide. We further confirmed the findings in the TRAMP mouse model, which is regarded as an adequate mouse model for NeCRPC. In the TRAMP model, with mice castrated at 12 weeks, nearly 80% of them developing NE-like carcinomas at 24 weeks [36, 37].

In conclusion, our results provide mechanistic insights into the development of NeCRPC. Our findings functionally reveal NTS-NTSR1/3 signaling in CK14^+^/CK8^+^ cells that provides a transdifferentiation advantage to NE-like cells in castrated microenvironments. Our findings also suggest that combined inhibition of both NTSR1 and AR could prevent the development of NED and prolong the duration of response to next-generation AR antagonists. This raises the possibility of clinically targeting NTS-NTSR1/3 axis both to limit the NED of cancer cells and to diminish castration resistance.

## Methods

### Fluorescence-activated cell sorting (FACS)

Whole tumors were dissected, cut into small pieces, and dissociated by using 0.5% collagenase Type III (Worthington Biochemical) and 1% Dispase II (Roche) in PBS for 1–2 h. Resulting single-cell suspensions were washed in PBS with 1% FBS and filtered through 70 mm nylon mesh. Cell fractions were incubated for 15 min at 4 °C with Fc block antibody (MACS) in PBS containing 1% BSA to avoid nonspecific antibody binding. Cells were subsequently washed in PBS/BSA and stained with either Ig controls or fluorophore-conjugated antibodies in MACS buffer (0.5% BSA, 2 mM EDTA in PBS). Data acquisition was performed on FACS Calibur (BD Biosciences) and analysis was done by using Flowjo version 9. The following antibodies were used: EpCAM-FITC (StemCell Technologies), CD45-PE, CD31-APC (WM59, BD PharMingen), and α-SMA-APC (BD-PharMingen).

### Immunohistochemistry of tissues

Formalin-fixed, paraffin-embedded (FFPE) sections were deparaffinized, blocked with 3% H_2_O_2_, and antigen retrieval was performed in 0.01 M citrate twice for 10 min in a microwave oven followed by a 60-min cool down. Slides were blocked with 3% normal goat serum, then incubated with various primary antibodies followed by Envision-plus-labeled polymer-conjugated horseradish peroxidase and DAB monitoring staining (Zhong Shan gold bridge, Beijing). Counterstaining was performed with Mayer-hematoxylin.

### Animal studies

Four-week-old male Babl/c mice were procured from a breeding colony at Chinese Academy of Sciences. 2 × 10^6^ LNCaP PCa cells suspended in 100 μl of PBS with 50% Matrigel (BD Biosciences) were implanted subcutaneously into the dorsal flank on both sides of the mice. Once the tumors reached an indicated stage, the animals were randomized without blinding and received castration or treatment of 10 mg kg^−1^ body weight MDV3100 or 25 mg kg^−1^ body weight SR48692 by oral gavage for 5 days per week (doses previously used in mouse PCa). Tumor volume was recorded by digital calipers and estimated using the formula (*π*/6) (*L* × *W*^2^), where *L* is length of tumor and *W* is width. At the end of the studies mice were killed and tumors were extracted and weighed. For TRAMP mice, we verified the genotypes by PCR using tail snip DNA as templates 42. Verified mice were randomized and received castration at 12 weeks or treatment of 10 mg kg^−1^ body weight MDV3100 or 25 mg kg^−1^ body weight SR48692 at 12 weeks by oral gavage for 5 days a week. At the end of the studies mice were killed and tumors extracted and weighed. All procedures involving mice were approved by the University Committee on Use and Care of Animals at the Tianjin Medical University and conform to all regulatory standards.

### Statistical analysis

The results were reported as mean ± SEM. For comparisons of central tendencies, normally distributed data sets were analyzed using two-sided Student’s *t*-tests under assumption of equal variance. Non-normally distributed data sets were analyzed using non-parametric Mann–Whitney *U*-tests. Time to disease relapse was estimated by the Kaplan–Meier method using GraphPad Prism 6 software. Unless indicated otherwise, the log rank test was used to assess statistical significance. All heatmaps were generated by the heatmap.2 function in the R package g-plots. Statistical tests were two sided; *P* < 0.05 was considered statistically significant.

## Supplementary information

Supplementary Information.

Supplementary Information.

Supplementary Information.

Supplementary Information.
