# Correction: Neurotensin and its receptors mediate neuroendocrine transdifferentiation in prostate cancer

**DOI:** 10.1038/s41388-019-0827-1

**Published:** 2019-05-02

**Authors:** Shimiao Zhu, Hao Tian, Xiaodan Niu, Jiang Wang, Xing Li, Ning Jiang, Simeng Wen, Xuanrong Chen, Shancheng Ren, Chuanliang Xu, Chawnshang Chang, Amilcar Flores-Morales, Zhiqun Shang, Yinghao Sun, Yuanjie Niu

**Affiliations:** 1 Department of Urology, Tianjin Institute of Urology, The Second Hospital of Tianjin Medical University, 300211 Tianjin, China; 2 University of Minnesota, Minnesota, MN 55455 USA; 3 Department of Urology, Changhai Hospital, Second Military Medical University, 200433 Shanghai, China; 4 Department of Pathology, University of Rochester, Rochester, NY 14620 USA; 5 Department of Health Science, Faculty of Health and Medical Sciences, University of Copenhagen, 2200 Copenhagen, Denmark

**Keywords:** Prostate cancer, Cancer therapeutic resistance

**Correction to: Oncogene**

10.1038/s41388-019-0750-5; published online 15 February 2019

The original version of this Article contained an error in the spelling of the author Amilcar Flores-Morales, which was incorrectly given as Amilcar Flores-Morelas. This has now been corrected in both the PDF and HTML versions of the Article.

