# Supplementary material for: Neurotensin and its receptors mediate neuroendocrine transdifferentiation in prostate cancer
Source: Oncogene. 2019 Feb 15;38(24):4875–84. doi: 10.1038/s41388-019-0750-5 (PMC6756221; doi:10.1038/s41388-019-0750-5)
Supplement: Supplementary file 4 — Supplementary Information. [file 41388_2019_750_MOESM4_ESM.doc]

**Supplemental Information for**

**Title:** **Neurotensin and its Receptors Mediate Neuroendocrine Transdifferentiation in Prostate Cancer**

**Contents**

**Supplementary Methods…………………………………………… p. 3-6**

**Supplementary Figures ……………………………………………. p. 7-17**

**Supplementary Figure 1 p. 7-8**

**Supplementary Figure 2 p. 9-10**

**Supplementary Figure 3 p. 11-12**

**Supplementary Figure 4 p. 13**

**Supplementary Figure 5 p. 14-15**

**Supplementary Figure 6 p. 16**

**Supplementary Refferences ………………………………………p. 17**

**Supplementary Methods**

**Primary Antibodies, Cell Culture and Reagents.**

NTS (Santa Cruze, sc-20806), Neuron-specific enolase (NSE, Abcam, ab53025), Chromogranin A (CgA, Abcam, ab15160), Synaptophysin (Syn, Abcam, ab8049), pan Cytokeratin (pan-CK, Abcam, ab6401), Achaete-scute homolog-1 (hASH-1, Abcam, ab74065, ab38557), repressor element silencing transcription factor (REST, Sigma-Aldrich, HPA006079), cytokeratin 8 (CK8, Abcam, ab9023) and cytokeratin 5 (CK5, Abcam, ab128190), P63 (Santa Cruz, sc-8431), AR (Santa Cruze, sc-816 and sc-7305), Erk1/2 (Cell Signaling Technology, #9102), Phospho-Erk1/2 (Cell Signaling Technology, #9101), NTSR1 (Sigma-Aldrich, SAB4502036), GFP (Abcam, ab6673), mCherry (Abcam, ab167453), NTSR2 (Abcam, ab48273), NTSR3 (Abcam, ab16640), Neuroblast differentiation-associated protein (AHNAK, Santa Cruze, sc-98373), BAT2 domain containing 1 (BAT2D1, Santa Cruze, sc-161375) and cAMP-dependent protein kinase catalytic subunit beta (PRKACB, Abcam, ab76238).

LNCaP, C4-2, PC3, HUVEC and THP-1 cells were obtained from the American Type Culture Collection. We maintained cell lines in RPMI 1640 (Gibco) supplemented with 10% Fetal bovine serum (FBS) (Gibco), 25 U/ml penicillin and 25 μg/ml streptomycin in a humidiﬁed 4% O2, 5% CO2, atmosphere. Human mast cell line (HMC-1) cells [^1^](#_ENREF_1) (kindly provided by Dr Joseph Butterfield, Mayo Clinic) were cultured in Iscove's Modified Dubecco's Medium (IMDM) (Hyclone), supplemented with 10% v/v FBS, 4.0 mM glutamine, 500 U/ml penicillin, 500 μg/ml streptomycin,125 μg/ml G418 (all from Invitrogen) and 0.01% v/v alphathioglycerol (Sigma). Primary fibroblast cells were generated by collagenase/hyaluronidase digestion from tumors. Primary fibroblast cells were cultured in MEGM medium (Lonza) supplemented with 10% FBS in a humidified 4% O2, 5% CO2, atmosphere. Neurotensin was purchased from Abcam, DMSO from Sigma. Alamar blue assays were performed using Resazurin (R&D Systems) following manufacturer’s procedures. Other antibodies were purchased from Abcam and chemicals were purchased from Sigma-Aldrich unless otherwise stated.

**shRNA and siRNA Knockdown studies**

The following genes were successfully knocked down using lentiviral vectors purchased from GeneCopoeia (Nts: MSH033327; NTSR1: HSH011959; NTSR2: HSH006276; NTSR3: MSH032227). Stable knockdown was achieved by selection with 1 mg/ml puromycin. Knockdown (>75%) was confirmed by western blot.

siRNA targeting the AHNAK gene (sc-97060), BAT2D1 (sc-88528), PRKACB (sc-39158) and all siRNA negative controls were purchased from Santa Cruz. Transfections were conducted using Lipofectamine RNAiMAX (Invitrogen).

**Confocal fluorescence analysis**

Cells were grown on Labtek II-CC2 Chamber slides (Nunc), fixed with 4% paraformaldehyde, and permeabilized with 0.2% Triton X-100/PBS prior blocking with 10% goat serum. Secondary antibodies were Donkey anti-mouse, -rabbit or -goat coupled to Alexa-350, -488 or -647 (Invitrogen). Cell nuclei were visualized with DAPI (Sigma).

**TUNEL Assay**

We purchased the Fluorescein-Frag EL DNA Fragmentation Detection Kit (Calbiochem), labeled FFPE sections following the manufacturer’s instructions, and counted the labeled nuclei by using a standard fluorescein filter at 465-495 nm.

**Immunoblotting**

Protein was extracted with RIPA lysis buffer. Protein lysates were resolved on an 8%-12% Bis-Tris Gel, transferred to PVDF membranes, probed with HRP-linked secondary antibodies (GE Healthcare), and visualized with ECL reagent (Thermo Scientiﬁc).

**ELISA**

Culture medium was collected following an incubation period of 48h and filtered through a 20-mm mesh (BD Falcon). ELISA for NTS was performed using commercially available kits according the manufacturer’s instruction (PHOENIX PHARMACEUTICALS, FEK-048-03). All ELISA readouts were normalized by cell number.

**Quantitative RT-PCR Analyses**

Total RNA was isolated from cells and tissues using TRIzol reagent (Invitrogen) in accordance with the manufacturer’s instructions. RT-PCR was performed on ABI 7900 96 HT series PCR machine (Applied Biosystem) or BioRad iCycler iQ (Bio-Rad Laboratories) using SYBR Green Supermix (Bio-Rad Laboratories). The gene-specific primer sets were used at a final concentration of 0.2 µM and their sequences are listed in **Supplementary Table 1**. All RT-PCR assays were performed in technical duplicate in at least three independent experiments using three different samples.

**Chromatin Immunoprecipitation**

CAFs cells were grown in DMEM (Invitrogen) supplemented with 10% charcoal-stripped FBS (CSF, HyClone, USA) for 48 h before stimulation with 1
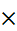
10^-9^ M R1881 or an equal volume of ethanol for 1 h. Chromatin immunoprecipitation (ChIP) was carried out using 5 μg of AR (N20, Santa Cruz) as previously described [^2^](#_ENREF_2).

**Immunohistochemical Analysis**

The use of human prostate cancer tissues and clinical data was approved by the Institutional Review Board of Tianjin Medical University. Informed consent was obtained from all patients. Serial tissue sections from human prostate cancer specimens were quantified in a blinded manner. The staining intensity and counts of positive cells were scored separately. Further details were provided in the Supplemental Experimental Procedures.

**Bioinformatic Analyses**

Gene expression profiles of xenograft tumors and NTS treated LNCaP cells were analyzed using Human Genome U133 Plus 2.0, following the manufacturer’s instructions. A total of 20 independent sets of biological replicates of CRLX samples and four sets of biological replications of Ctrl were used. Among the 20 independent sets of CRLX samples, significantly evaluated NTS were found in 8 sets. Signatures of CRLX included genes that were over- or low-expressed in the 8 sets of xenograft tumors from CRLX compared to Ctrl. Differentially expressed genes between CRLX and Ctrl were clustered by k-means clustering. The gene expression analysis was performed as follows: out of 22,288 genes on the array, only those genes whose expression varied by at least one standard deviation from the mean across all conditions were included. In addition, genes with significant variation between duplicates (p < 0.05 in a student’s t test) were excluded. A total of 589 genes were left, clustered by k-means clustering (Table S2).

To seek out the optimal concentration of NTS in vitro, Gene Set Enrichment Analysis (GSEA, http://www.broadinstitute.org/gsea/) was performed using overexpressed genes in CRLX signature as the gene set, NTS-regulated genes as the phenotype data. Statistical significance was assessed by comparing the enrichment score to enrichment results generated from 1,000 random permutations of the gene set to obtain P values (nominal P value).

For principal component analysis (PCA), we first performed Z-transformation for each gene in each individual data set, using the formula Zi = (Xi-mean(X))/sd(X), where Zi represents the z-score of the ith sample, Xi is the expression value of this gene, and mean (X) and sd (X) represent the average value and standard deviation of the ith gene across all samples, respectively. The results were plotted using the first three principal components using ‘‘scatterplot3d’’ package of R.

Correlation between NTS (probe set: 206291_at) and other genes was measured as the mean of Pearson’s correlation coefficients from the independent 20 gene array data sets of CRLX. Focusing on G-protein coupled receptor signaling and neurogenesis, including neuronal differentiation and synaptogenesis. Genes with intracellular function were selected by filtering out genes that did not belong to the Gene Ontology Category Intracellular Space (GO:0043232).

**Supplementary Figures**

**
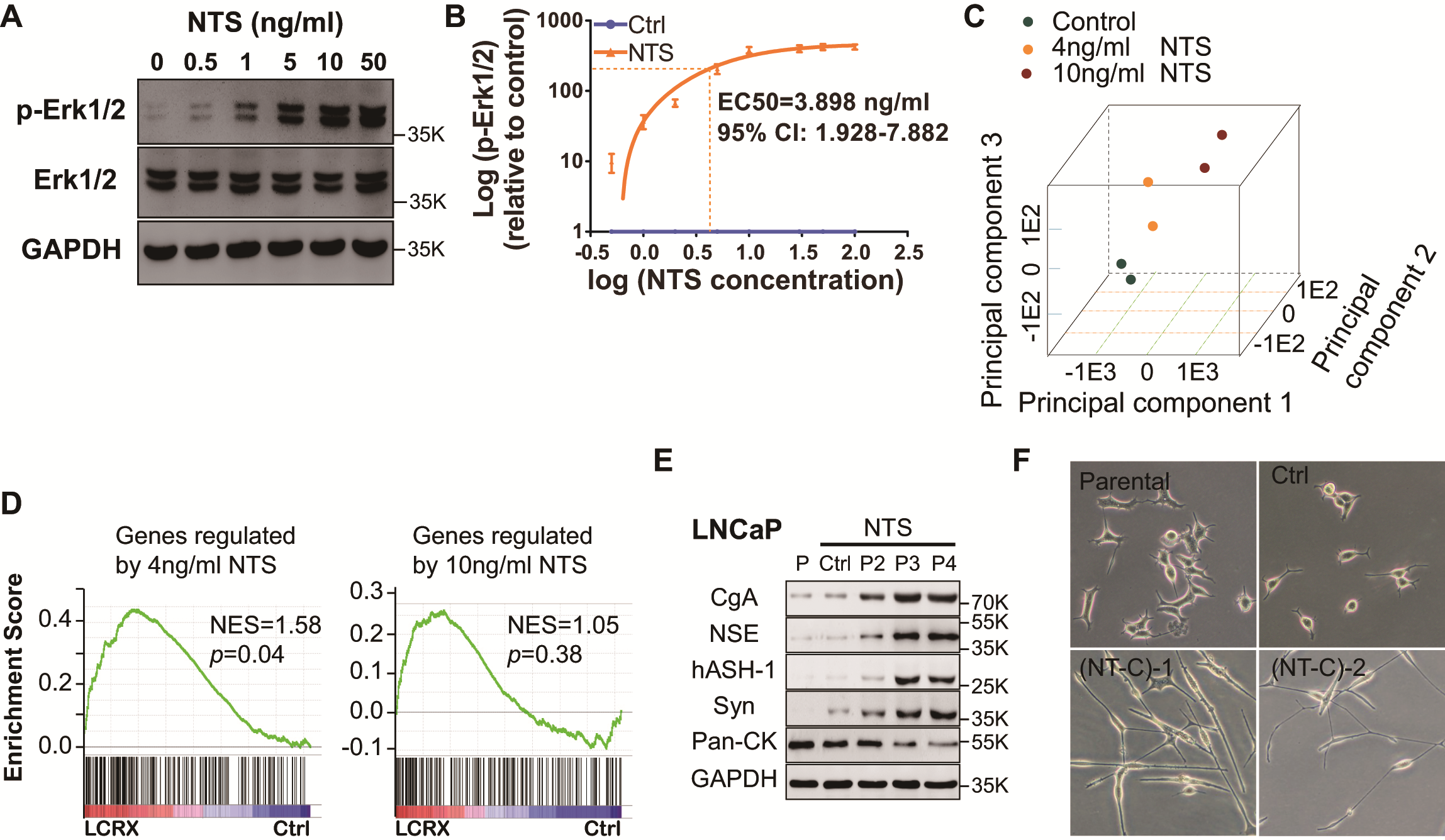
**

**Supplementary Fig. 1 Permissive concentration of NTS for NED**

(A) Western immunoblotting analysis of Erk1/2 activation in response to different concentrations of NTS, in LNCaP cells.

(B) EC50 represents the NTS concentration yielding half-maximal activation of Erk1/2, as determinated in (A). DMSO was used as negative control.

(C) Principal component analysis of gene expression profiles of LNCaP cells treated with NTS at EC50 (4 ng/ml) and saturating (10 ng/ml) concentrations is presented. The first three principal components are shown.

(D) GSEA of association between gene sets induced by NTS at the two concentrations and CRLX signature status in LNCaP xenograft tumors (See also Table S2) is shown.

(E) Immunoblotting analysis of CgA, NSE, Syn, hASH1 and pan-CK in cells of each group from different passages.

(F) Bright field microscopy: parental, NTS (4ng/ml)-treated and DMSO-treated C4-2 cells.


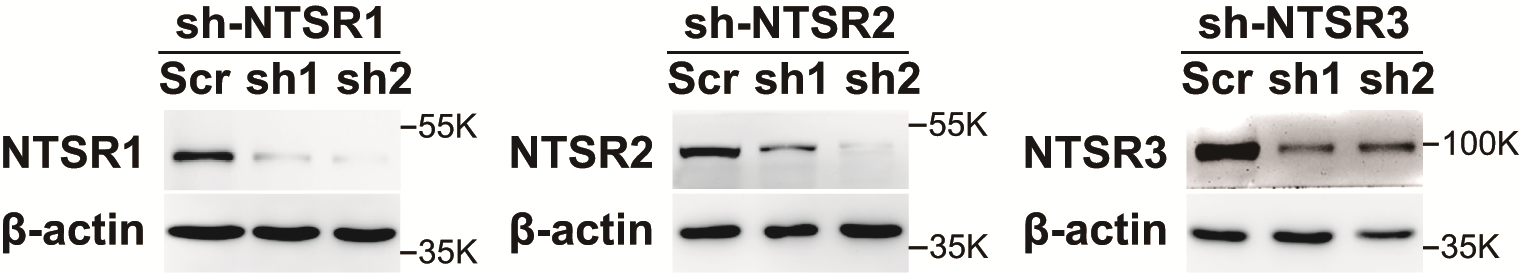


**Supplementary Fig. 2 NTSR1 and NTSR3 are required for NTS-stimulated NED in LNCaP cells**

The efficiency of sh-NTSR1, sh-NTSR2 and sh-NTSR3 were determined by immunoblotting analyses of NTSR1, 2 and 3 expressions in CK8^+^/CK14^+^ cells. Two independent short hairpin RNAs targeting each NTSR were tested.


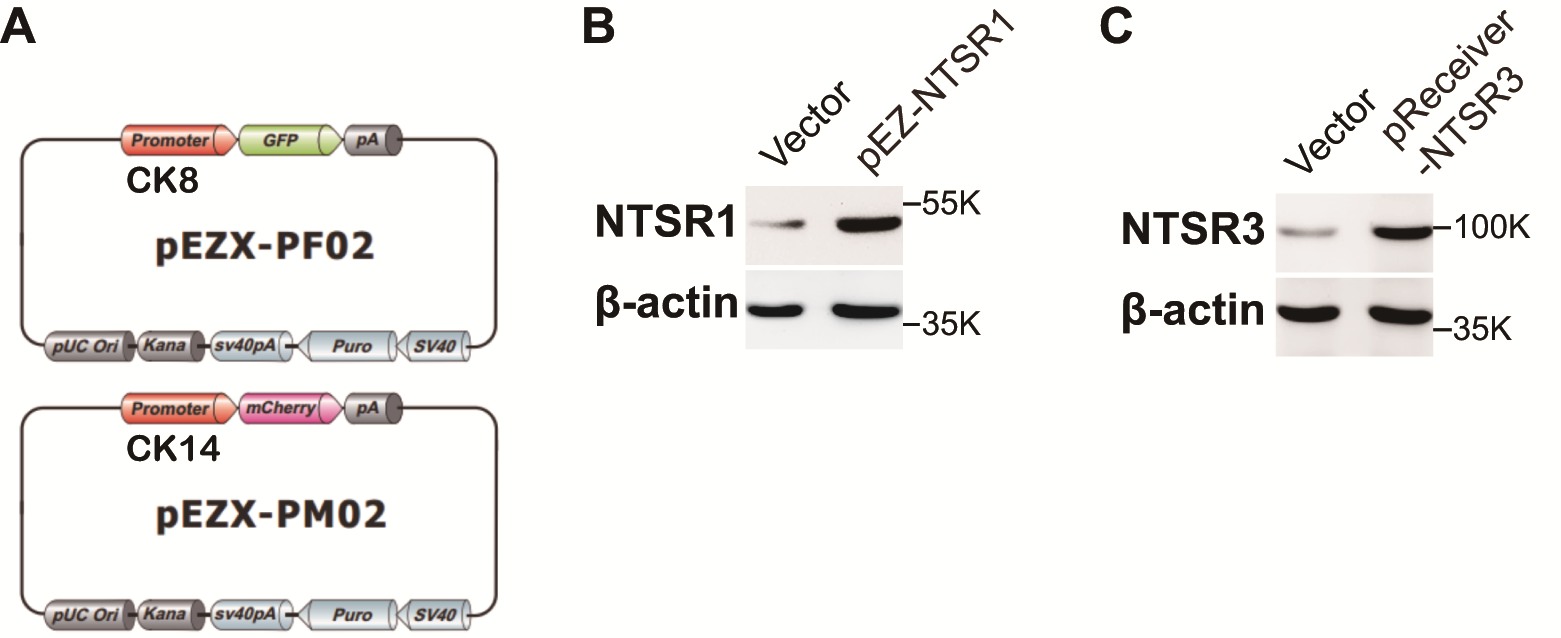


**Supplementary Fig. 3 CK8^+^/CK14^+^ Cell population to be the candidates of NED**

(A) Diagram of the lentiviral reporter system.

(B) The efficiency of pEZ-NTSR1 was determined by immunoblotting analyses of NTSR1 expression in CK8^+^/CK14^-^ cells.

(C) The efficiency of pReceiver-NTSR3 was determined by immunoblotting analyses of NTSR3 expression in CK8^-^/CK14^+^ cells.


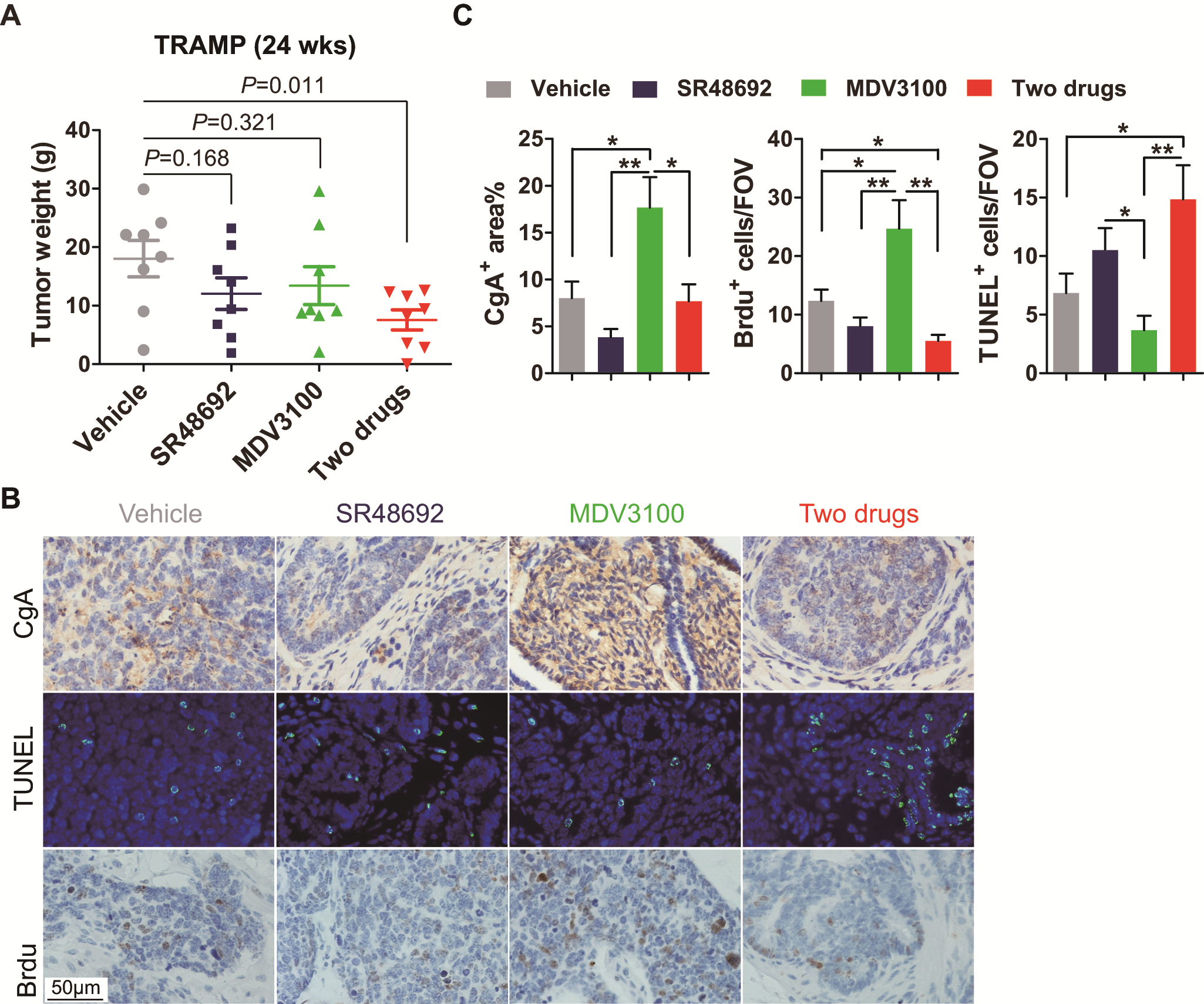


**Supplementary Fig. 4 Suppression of NED and castration resistance by NTSR1 antagonist**

(A) 16wks old TRAMP mice were randomized and then received PEG vehicle, 25mgkg^-1^ SR48692, 10 mgkg^-1^ MDV3100 and SR48692+MDV3100 for 5 days a week. The prostate tumors were harvested at 24wks. n=8 mice per group.

(B) 16wks old TRAMP mice were randomized and then received PEG vehicle, 25mgkg^-1^ SR48692, 10 mgkg^-1^ MDV3100 and SR48692+MDV3100 for 5 days a week. Representative images of Ki-67, TUNEL and CgA in TRAMP tumors from each group.

(C) Quantitation of Ki-67, TUNEL and CgA in TRAMP tumors from each group at 24wks. Immunostained area/FOV was quantified using ImageJ. *P* values were evaluated by Mann-Whitney U test.


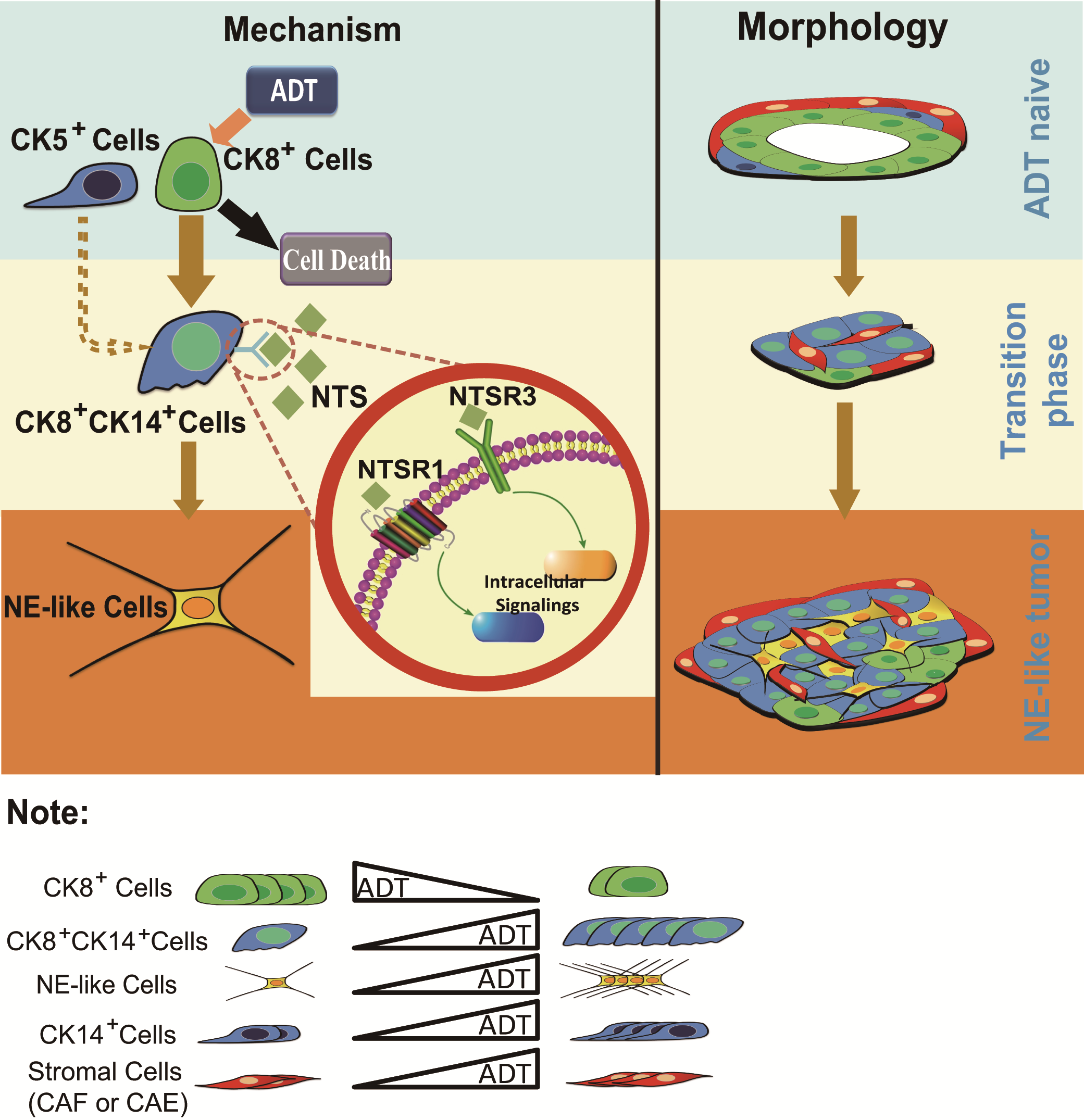


**Supplementary Fig. 5 Graphical Abstract**

Schematic depicting the mechanism of NTS networks induces NED in prostate cancer after ADT. ADT results in the death of luminal cancer cells and simultaneously increase NTS production in tumor. Subsequently, the NTS induces CK5^+^/CK8^+^cells to NE-like cells via NTSR1 and NTSR3.

**Supplementary Refferences**

1 Butterfield, J. H., Weiler, D., Dewald, G. & Gleich, G. J. Establishment of an immature mast cell line from a patient with mast cell leukemia. Leukemia research 12, 345-355 (1988).

2 Massie, C. E. et al. New androgen receptor genomic targets show an interaction with the ETS1 transcription factor. EMBO reports 8, 871-878, doi:10.1038/sj.embor.7401046 (2007).
